# Mapping the Abundance and Distribution of Adélie Penguins Using Landsat-7: First Steps towards an Integrated Multi-Sensor Pipeline for Tracking Populations at the Continental Scale

**DOI:** 10.1371/journal.pone.0113301

**Published:** 2014-11-20

**Authors:** Heather J. Lynch, Mathew R. Schwaller

**Affiliations:** 1 Department of Ecology and Evolution, Stony Brook University, Stony Brook, New York, United States of America; 2 Mesoscale Atmospheric Processes Laboratory, NASA/Goddard Space Science Center, Greenbelt, Maryland, United States of America; University California Los Angeles, United States of America

## Abstract

The last several years have seen an increased interest in the use of remote sensing to identify the location of penguin colonies in Antarctica, and the estimation of the abundance of breeding pairs contained therein. High-resolution (sub-meter) commercial satellite imagery (e.g., Worldview-1, Quickbird) is capable of colony detection and abundance estimation for both large and small colonies, and has already been used in a continental-scale survey of Adélie penguins. Medium-resolution Landsat imagery has been used successfully to detect the presence of breeding penguins, but has not been used previously for abundance estimation nor evaluated in terms of its minimum colony size detection threshold. We report on the first comprehensive analysis of the performance of these two methods for both detection and abundance estimation, identify the sensor-specific failure modes that can lead to both false positives and false negatives, and compare the abundance estimates of each method over multiple spatial scales. We find that errors of omission using Landsat imagery are low for colonies larger than ∼10,000 breeding pairs. Both high-resolution and Landsat imagery can be used to obtain unbiased estimates of abundance, and while Landsat-derived abundance estimates have high variance for individual breeding colonies relative to estimates derived from high-resolution imagery, this difference declines as the spatial domain of interest is increased. At the continental scale, abundance estimates using the two methods are roughly equivalent. Our comparison of these two methods represents a bridge between the more developed high-resolution imagery, which can be expensive to obtain, and the medium-resolution Landsat-7 record, which is freely available; this comparison of methodologies represents an essential step towards integration of these disparate sources of data for regional assessments of Adélie population abundance and distribution.

## Introduction

Though the idea of using satellite imagery to map penguin colonies is over thirty-years old [1], the use of these approaches for ecological research and long-term monitoring has been in active development only in the last few years (e.g., [2]–[4]). Recent data policy changes, such as the free availability of archival Landsat imagery, have opened access to vast amounts of medium resolution satellite imagery over Antarctica, and while access to very high-resolution (VHR) imagery is hampered by licensing and cost, increased tasking of satellites for imagery in the Antarctic have rapidly increased the quantity of imagery available. Recent studies demonstrate that penguin colony presence and abundance can be detected on a continental scale by manual interpretation of very high resolution (VHR) satellite imagery [5] and that automated algorithms that exploit the spectral signature of penguin guano can be used to detect the presence of penguin colonies in medium-resolution Landsat data [2], [6]. Taken together, these new analytic methods—and the Terabytes of data to which they can be applied—afford the possibility of routine, continent-wide surveys of Adélie penguin presence and abundance, although to date no attempt has been made to use Landsat for regional abundance estimation or to compare the detection and abundance information derived from these two types of satellite images. Our analysis represents a bridge between abundance estimates based on very high-resolution imagery and the medium-resolution data record available from Landsats-4, -5 and -7. Forging this connection will facilitate integrated abundance estimates in the era when both of these satellite data types overlap, and will advance the development of a continuous time series of abundance that leverages a Landsat data record dating back to the 1980s.

This paper presents the results of an automated retrieval of Adélie penguin colonies along the entire continent of Antarctica from medium-resolution Landsat-7 imagery (30 m pixel size), and its comparison with data on presence and abundance based on manual interpretation of VHR satellite imagery (∼0.60 m pixel size). Landsat-7 launched on April 15, 1999 and has collected imagery over Antarctica continuously since that time. Unfortunately, the ETM+ Scan Line Corrector failure in 2003 occurred just as some of the first very-high resolution images became available for the Antarctic, precluding direct contemporaneous comparison of abundance estimates from these two types of sensors. We leverage the recently published global Adélie survey [5] to assess whether Landsat-7 can be used to estimate abundance as well as occupancy, and to estimate the lower detection threshold for Landsat-7. Since the uncorrupted Landsat-7 imagery dates from 1999–2003 and the global Adélie survey [5] estimates predominately stem from 2010–2013, the correlation between the abundance estimates will be highly conservative, since the correlation is degraded by changes in true abundance in the intervening decade. Nevertheless, the relatively slow dynamics of penguin colonies means that abundances between these two periods should be fairly consistent within the margin of error of the abundance estimation itself, allowing us to investigate whether the number of pixels retrieved in the earlier period scales with the abundance reported by [5] for the latter period. This cross-validation approach is the best currently available to quantify the accuracy and identify sources of error in using medium-resolution Landsat imagery for occupancy (presence vs. absence) and abundance. Controlling or correcting these errors is a key consideration for future automated surveys of Adélie penguins in Antarctica.

In our analysis and discussion, we explicitly distinguish between the probability of detection and the estimation of abundance conditional on the detection of a colony. The detection of a colony based on the spectral characteristics of its guano stain is a binary classification subject to errors of omission (false negative) and commission (false positive). Abundance estimation is a separate challenge in which success is judged by the accuracy (bias and variance) of estimates of population size. We use the detection probability model along with a probability model for colony size to estimate the total abundance of penguins missing given the results of a Landsat survey. This methodology allows us to correct future Landsat surveys for detection failures and paves the way for achieving regular unbiased estimates of the global Adélie population. We considered continental Antarctica (in which the Adélie penguin is the only breeding *Pygoscelis* spp. penguin) separate from the Antarctic Peninsula in our analysis. Guano stains on the Antarctic Peninsula are ambiguous with respect to species in Landsat imagery and the three *Pygoscelis* spp often nest in colonies mixed at Landsat's larger spatial resolution. Additionally, as described in the Discussion, these two regions have different ‘failure modes’ with respect to detection and imagery interpretation.

Our goals for this analysis were three-fold: (1) To estimate the lower detection threshold for Landsat-7 imagery, (2) to demonstrate that the number of pixels classified as penguin guano in Landsat-7 scales with abundance, and (3) to estimate variance in abundance estimates at varying scales of spatial aggregation. Our survey includes both continental Antarctica, where Landsat imagery has been used for detection of Adélie penguin colonies previously [2], as well as the Antarctic Peninsula, where its use is novel. The comparison and cross-validation of multiple data streams is a necessary step in the full integration of these data into a single remote sensing retrieval and analysis pipeline for monitoring Adélie penguin populations. This approach represents a significant improvement over direct field counts alone, which lack the scalability of remote sensing surveys, and are subject to underestimation bias in regional or global estimates due to the remoteness and inaccessibility of sections of the Antarctic coastline [7]. Population estimates that scale in space and time are expected to more seamlessly interoperate with trophic and climate models, so that changes in Adélie penguin populations can be clearly tied to harvesting in the Southern Ocean fisheries and climate change.

## Methods

### Automated Landsat retrievals

Retrieval of Adélie penguin colony location and spatial extent over the Antarctic Peninsula was conducted using Landsat-7 Enhanced Thematic Mapper-Plus ETM+ data. The Landsat data used in this study were obtained as a Climate Data Record (CDR), which was generated using the methods developed by [8], and distributed by the U.S. Geological Survey Earth Resources Observation and Science Center (https://espa.cr.usgs.gov). This CDR applies a uniform processing to the entire dataset, and includes a host of products including top of atmosphere (TOA) reflectance, surface reflectance, brightness temperature, as well as masks for clouds, cloud shadows, adjacent clouds, land and water. The Landsat CDR TOA product was used for the automated retrieval of Adélie penguin colonies following the methods described in [2]. One difference between our Landsat survey and that described in [2] is that additional exposed soil and rock surfaces were sampled and included in the training dataset for the AP retrieval algorithm because the AP has a proportionally larger expanse of exposed soil and a different suite of soil types than the rest of the continent. The training dataset consisted of 473 Landsat-7 pixels selected from known Adélie colonies plus an additional 10,688 pixels selected from areas of exposed rock, soil and vegetation. The retrieval algorithm used this training dataset to define an ellipsoid in the spectral data space that surrounds pixels from Adélie colonies, with the assumption that the surface of the ellipsoid separates the pixels within its bounds from other surface types. The equation of the ellipsoid is captured in a “transition matrix” which is used to build a decision rule for determining whether or not new pixels belong to the Adélie colony class. All of the Landsat-7 ETM+ reflective bands (bands 1–5 plus band 7) were used in the AP retrieval. The retrieval on the AP and surrounding islands was conducted on 62 scenes covering this region but did not include the South Shetlands or the South Orkney Islands because cloud-free imagery was unavailable for the era investigated. The methods described, however, can be directly applied to imagery from these areas in future surveys. Additional details on the AP retrieval algorithm, including the transition matrix and decision rules used in this study, can be found in the additional information within the file Table S2. An Antarctic continent-wide retrieval of Adélie penguin colonies was obtained by combining the AP retrievals with those previously generated by [2]. In all cases, imagery was selected from the Landsat-7 era in the years from 1999–2003, prior to the failure of the ETM+ Scan Line Corrector.

### Abundance and distribution from high-resolution commercial satellite imagery

We used the Adélie penguin survey by [5], which was based on VHR imagery supplemented with recent field surveys, as the basis for studying the performance characteristics of Landsat retrievals. Details of the detection and abundance estimation of Adélie penguins in VHR imagery may be found in [4] and [5]. From their global survey of Adélie penguins, [5] produced a detailed list of all Adélie penguin colonies (location and estimated abundance) known to exist at the time of the survey, which we used as a baseline for assessing successful retrieval and abundance estimation using the lower resolution Landsat survey method. The comparison of our automated detection algorithm to the Adélie survey results from [5] yields four possible outcomes for each potential Adélie penguin breeding site.

1. A penguin colony is detected using both methods.
2. No penguin colony is detected by either approach. This situation encompasses the vast majority of the Antarctic coastline.
3. A penguin colony is identified in [5] but not identified by our automated detection algorithm applied to Landsat imagery. These incidents are considered errors of omission for the Landsat detection algorithm because the colonies identified by [5] were, with a few exceptions, validated by direct field surveys. In these cases, we consulted the available imagery to determine what characteristics may have led to this error of omission.
4. An area is flagged as containing a colony of breeding penguins by the automated detection algorithm but was not reported in [5]. These cases may represent an error of omission for [5] or an error of commission by the automated detection algorithm. In these cases, we searched for additional VHR imagery that might resolve these two possible scenarios. If no additional imagery was available, we were not able to determine the nature of the discrepancy. All such cases are reported in the hopes that future field surveys can validate the true occupancy status of these locations.

The conversion between sensor pixels flagged as guano and the abundance of penguins breeding within that area is determined by an ‘apparent density’ that is not necessarily equal to the actual density of breeding pairs as measured on the ground [4]. Nevertheless, it has been demonstrated that the closely packed nature of Adélie penguin colonies allows for the estimation of abundance based on the area of guano staining in very high-resolution imagery [4]. As a first pass, we used Kendall's 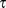 rank correlation coefficient to investigate whether the number of pixels retrieved by Landsat-7 scales with penguin abundance as reported by [5]. In other words, we ask whether the colony with the largest number of pixels retrieved by Landsat-7 is also the largest colony as reported in [5], and so forth. We then use the abundance estimates of [5] to develop a predictive model for estimating the total abundance of Adélie penguins from medium-resolution Landsat imagery, recognizing that the ∼10 year time lag between the abundance estimates reported in [5] and the older Landsat imagery significantly increases the uncertainty of these estimates. Nevertheless, such ‘ballpark’ estimates can be invaluable in the near term, particularly for estimating approximate abundance for previously undiscovered colonies, and provide a road map for re-analysis when matched-pairs of Landsat-8 imagery and field counts become available in the future. This methodology, applied here to Landsat-7 imagery and in the future to Landsat-8 or other medium resolution sensors, can be used to automate future continental scale surveys yielding global estimates of Adélie distribution and abundance with little to no manual interpretation. This abundance model involves two components: (1) the estimation of breeding abundance represented by the pixels identified as guano in the Landsat survey, and (2) the estimation of the abundance of breeding Adélie penguins not detected.

### Abundance estimation for detected colonies

For the first component of total abundance, involving the estimation of abundance conditional on detection, we considered all breeding populations found by the Landsat-7 automated retrieval process whose population had been estimated previously by [5]. The data used for our abundance estimates are detailed in Table S1. Seven colonies were not included in the development or validation of the abundance model because they either included a significant number of flying birds (which inflated the estimated size of the Adélie colony) or because a discrepancy between the Landsat retrieval and the location reported in [5] made it impossible to know whether the same breeding population was being considered in each method (Table S2). We used a Poisson regression model for abundance as a function of guano area



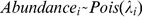
(1)

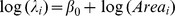
where 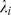 is the expected abundance of the *i* ^th^ colony and e 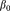 can be interpreted as an “apparent density” (nests per ‘detected guano area’). Since many applications, particularly within the context of the Convention on the Conservation of Antarctic Marine Living Resources, require abundance estimates at scales larger than an individual breeding colony (e.g., Convention Subareas), we also quantified predictive accuracy of our final abundance model for spatial units of increasing scale, from individual colonies to the entire continent.

### Estimation of abundance in colonies not detected by Landsat

The total abundance of Adélie penguins not detected in a Landsat survey is a convolution of the probability distribution of colony abundance and the probability of detecting a colony given (i.e. conditional on) its size. The expected size of a colony that is not detected by Landsat imagery can be calculated as the following conditional expectation
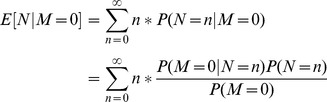
(2)where *N* is the number of breeding pairs, and *M* represents the binary probability that a colony is missing (M = 0) from a Landsat survey. The probability that a colony is missing conditional on its size (i.e., *P(M = 0|N = n)  =  1-detection probability*) can be estimated with a logistic regression model fit using the abundances of each colony as reported in [5]. In the absence of future matched VHR-Landsat surveys, the detection probability can be assumed a function of the sensor that would not change for future surveys using Landsat-7 imagery. The probability distribution of colony sizes *P*(*N*) is a long-tailed distribution best fit by a log-normal distribution. In theory, we could estimate the best-fit parameters for the colony size probability distribution *P*(*N*) using the partially-censored Landsat survey detections by inverting Eq. 2 for *P*(*N*); however, the low rates of detection for very small colonies make it difficult to establish the shape of the colony size distribution from the Landsat detections alone. We therefore fit a log-normal distribution to the abundance data in [5] and used a discretized version of this best-fitting model for *P*(*N*) in Eq. 2 to estimate the total abundance of Adélie penguins missing from the Landsat survey due to non-detection.

The sum in Eq. 2 can be calculated using Markov Chain Monte Carlo integration, and the resulting expectation can be multiplied by expected number of missing colonies to estimate the total missing abundance. We modeled the total number of undetected Adélie colonies in the surveyed area as a sample from a Negative Binomial distribution



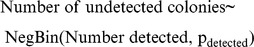


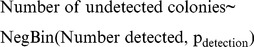
(3)where the overall probability of detection 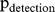 was estimated using the survey data in [5] as a basis for comparison.

### Estimation of abundance at aggregated spatial scales

To estimate the improvement in predictive accuracy at regional scales containing multiple penguin colonies, we compared total abundance estimated by VHR and Landsat for subsets of the original data. Our goal was to determine how many colonies 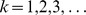 would have to be enclosed within an area for the estimate of total abundance by Landsat-7 to be statistically indistinguishable from the corresponding total abundance estimated by VHR. For example, to compare total abundance estimates for a (hypothetical) area containing *k* = 5 colonies, we would draw 5 colonies at random from the total set of colonies in our study (continental and Peninsula areas combined) and use Eq. 1 (with best-fit parameters; see Results) to estimate the total abundance that would be estimated for those 5 colonies. The total abundance would then be compared to the expected total abundance from the VHR survey of [5], and the scaled difference recorded as



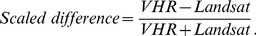
(4)Repeating this procedure 1000 times, we generated a distribution of scaled differences between the two methods. The null distribution consisted of calculating the same scaled difference but with the Landsat abundance replaced by a second independent draw from the VHR abundance distribution. (The abundance of each colony is associated with a sampling distribution, so independent draws from that distribution yield different estimates of abundance. This variability represents our uncertainty in the true abundance of the colony.) In other words, we wanted to differentiate between differences due to Landsat's relatively poorer performance characteristics, and differences due to inherent uncertainty for the VHR counts used as “true” abundance. The empirical cumulative distribution functions (VHR-Landsat vs. VHR-VHR) were compared using a one-sided Komolgorov-Smirnov test.

## Results

Using Landsat data over the Antarctic Peninsula, the automated retrieval flagged 143 areas as belonging to the “Adélie penguin colony” class. Based on the site-by-site comparisons described above, these retrievals included 16 confirmed Adélie penguin colonies. Among these retrieved areas were 17 additional areas identified as likely Adélie penguin colonies that could not be confirmed because VHR data were not available and the sites were not previously mentioned in the literature (see Table S3). Using Landsat data over continental Antarctica, the automated retrieval flagged 187 areas as belonging to the “Adélie penguin colony” class, within which were 91 confirmed Adélie penguin colonies, and 8 areas identified as likely, but unconfirmed, Adélie penguin colonies (see Table S3). The Landsat retrieval of Adélie penguin colonies over the entire continent of Antarctica can be quickly visualized via the gzip-compressed Keyhole Markup Language (kmz) formatted file provided (see additional information within the file Table S2).

### Probability of detection

**Figure 1:**
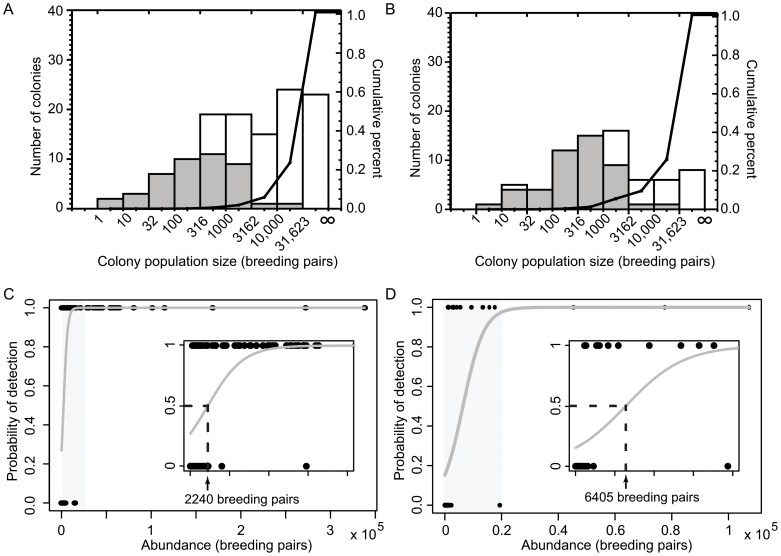
Probability of detection using Landsat-7 imagery. (A, B) The number of Adélie penguin colonies located (white) and missed (shaded) by the Landsat retrieval method for continental Antarctica (A) and the Antarctic Peninsula (B). The percent contribution of each bin to the total population is also illustrated. The horizontal axis is logarithmic, with boundaries equal to 10^0^, 10^1^, 10^1.5^, 10^2^, 10^2.5^, etc. (C, D) Probability of detection as a function of colony size (as reported by [5]) along the coast of continental Antarctica (C) and on the Antarctic Peninsula (D). Gray line represents best-fitting logistic model. Inset represents portion of the plot shaded in light gray.

The detection performance of the automated Landsat retrieval improved with increasing Adélie penguin colony population size (Figure 1). The probability of detecting an Adélie penguin colony using Landsat (*p_detection_*) was modelled using the logistic transformation
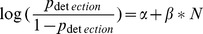
(5)where N is the abundance of breeding pairs, 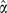 = −1.00 (SE = 0.32) and 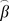 = 0.0004 (SE = 0.0001) for the continental colonies and 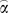 = −1.72 (SE = 0.45) and 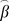 = 0.0003 (SE = 0.0001) for the Peninsula colonies. The smallest colony detected in the Landsat retrieval had 322 pairs (Clark Island in Marie Byrd Land), but the threshold for 50% detection probability (as estimated by the logistic model) was 2240 breeding pairs for continental colonies and 6405 breeding pairs for Peninsula colonies (Table 1).

**Table 1:** Probability of detection using Landsat-7 retrieval algorithm.

p_detection_	Colony size	
	Continent	AP
0.50	2240	6405
0.75	4705	10485
0.90	7190	14575
0.95	8870	17355
0.99	12590	23495

Colony sizes (breeding pairs) at various probabilities of detection using Landsat-7 retrieval algorithm.

While nearly all of the smallest colonies go undetected using the automated interpretation of Landsat imagery, small Adélie penguin colonies (1 to 3000 pairs) contribute only ∼2% to the total Adélie population [5]. Along the coastline of continental Antarctica, the Landsat method successfully identified Adélie penguin colonies making up 98.3% of the population total for this region. The AP has a significantly larger proportion of small colonies when compared to the rest of the continent, and consequently the Landsat method missed a larger number of these colonies in both absolute numbers and in proportion to the total (Figure 1). On the AP, the colonies found by the Landsat method account for 90.2% of the sample population. When all data are pooled together, the Landsat method retrieved those colonies that account for 97.3% of the population total. Furthermore, the Landsat retrieval algorithm detected a number of potential Adélie colonies not captured by the manual imagery search of [5], see Table S3.

There were three Adélie colonies with populations greater than 4000 pairs that were missed in the Landsat retrievals: on Haswell Island (4011 pairs), Coulman South (14786 pairs), and Penguin Point (19377). As described in [2] it is likely that Haswell Island was abandoned during the period when the Landsat imagery were collected (2001 and 2002). The Coulman South colony was covered by deep shadow in the Landsat imagery and is therefore a true error of omission. The only useable Landsat imagery over Penguin Point available during the survey era was collected on November 24, 2001, which is very early in the Adélie penguin breeding season. A second cloud-free image from January 8, 2001 was available but it was unusable due to band saturation. It is likely that cloud-free Landsat imagery from later in the breeding season would resolve the Penguin Point colony (Figure S1).

### Abundance estimation for detected colonies

**Figure 2:**
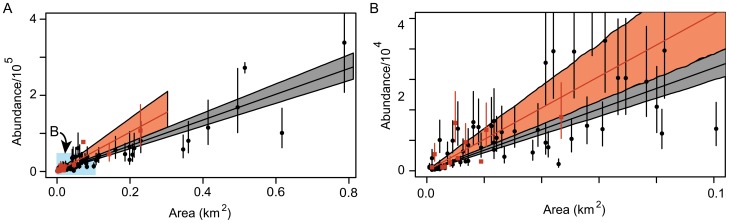
Colony abundance vs. guano stain area. (A) Colony abundance (and 95^th^ percentile confidence intervals; as reported by [7] as a function of the area identified as guano in the Landsat-7 survey (black circles  =  continental Antarctica, orange squares  =  Antarctic Peninsula), with best-fitting Poisson regression model (and associated 95^th^ percentile prediction envelop; gray-shaded envelop  =  continental Antarctica, orange-shaded envelop  =  Antarctic Peninsula). (B) Zoomed in portion shown as blue box in Panel A.

The number of Landsat-7 pixels retrieved as penguin guano is significantly correlated to the abundance of breeding penguins as reported in [5] (Kendall's 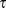 = 0.73 [n = 88; p<0.001]). The Poisson regression model in Eq. 1 yielded estimates of 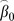 = −1.0844 (SE = 0.0007) and 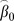 = −0.661 (SE = 0.002), for the continental and Peninsula data, respectively, corresponding to estimates of apparent density of 0.34 nests/m^2^ and 0.52 nests/m^2^, respectively. As expected from a Poisson regression, the predictive envelope grows with colony size (Figure 2).

### Estimation of abundance of colonies not detected by Landsat

**Figure 3:**
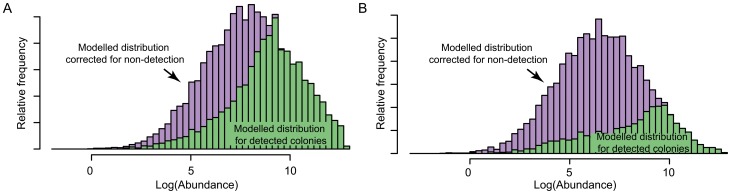
Colony size distribution censored by Landsat-7 detection limit. We used a log-normal distribution model for colony size to calculate the total abundance of Adélie penguins not captured by our Landsat survey (see Eq. 2). The influence of size-dependent non-detection on the distribution of colony sizes detected by Landsat in continental Antarctica (A) and on the Antarctic Peninsula (B) can be seen in the difference between the modelled distribution of colony sizes (log-scale) for all Adélie penguin colonies (purple) and the distribution of colony sizes as detected by Landsat (green).

We used the distribution of colony abundances reported in [5], to find the best estimates of the log-normal distribution parameters for *P*(*N*) (Figure 3)
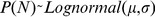
(6)and found 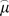 = 8.06 (SE = 0.21) and 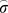 = 2.34 (SE = 0.15) for the continental colonies and 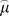 = 6.64 (SE = 0.32) and 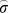 = 2.19 (SE = 0.23) for the Peninsula colonies. Using Eq. 2, we estimate the total (Peninsula + continent) number of Adélie breeding pairs missing from the Landsat survey to be 119738 (95^th^ percentile CI: 70364–188070), which is higher but generally consistent with the empirical value of 75582 breeding pairs found in the VHR survey [5] but not found by Landsat-7.

### Estimation of abundance in colonies with no prior estimates

The Landsat survey found eight colonies that were not detected in the VHR survey reported by [5] but which are outside the breeding range of either gentoo penguins (*Pygoscelis papua*) or chinstrap penguins (*P. antarcticus*), which can appear similar in satellite imagery [9]. While other errors of commission are possible, we can be confident that these are not conflated with mis-identification of penguin species. Using Eq. 1, we estimate the total abundance of Adélie penguins in these eight colonies as ∼38000 breeding pairs (Table S3), although these estimates, and in fact the existence of Adélie penguins at these eight locations, needs to be confirmed by higher-resolution satellite imagery or direct observation.

Table S3 lists 17 additional locations flagged as penguin colonies by the Landsat method that are within the breeding range of all three *Pygoscelis* species and thus cannot be identified to species, three of which are large penguin colonies in the Danger Islands. The Danger Islands, a collection of islands off the tip of Joinville Island at the end of the Antarctic Peninsula, are poorly surveyed due to frequent heavy ice cover and steep terrain that preclude easy access, but are known to contain extremely large populations of Adélie and gentoo penguins [10], [11]. Within the Danger Islands, the Landsat survey identified three major penguin populations at Brash Island, Earle Island, and Darwin Island, containing an estimated 166078 (95^th^ percentile CI: 123666–228268), 23649 (95^th^ percentile CI: 17361–32163), and 7419 (95^th^ percentile CI: 5384–9931) breeding pairs of penguins, respectively. Based on prior surveys of the area, these are likely to be overwhelmingly composed of Adélie penguins, making Brash Island potentially one of the largest Adélie penguin colonies in the world.

Jorge Island was identified by [10] as one location for which no census information existed, and was another location identified by the Landsat survey as containing breeding penguins. We estimate 3733 (95^th^ percentile CI: 2710–5064) breeding pairs of penguins (of unknown species) breeding at this location.

### Errors of commission

An earlier study [2] of Adélie penguin detection in continental Antarctica reported commission errors for the Landsat method on the order of 1% or less in the retrieval of Adélie colonies along the southern coastline of Antarctica. In a similar analysis for the retrievals on the Antarctic Peninsula, we used required prior knowledge of these locations and, where possible, confirmation by high-resolution satellite imagery. We find that commission errors for the Antarctic Peninsula fall into several categories including: pixels that identify potentially new colonies as determined by visual examination of high-resolution satellite imagery but which have not yet been confirmed by other methods, pixels that identify other penguin colonies or those with mixed species including Adélie penguins, pixels that identify flying bird breeding areas (known to exist, or previously unknown to exist), and pixels that are “pure errors” where various soil types are incorrectly categorized as Adélie penguin colonies. The commission error for soils incorrectly classified as penguin colony was calculated to be 3% for the Antarctic Peninsula, and 2% for the entire continent.

### Aggregated estimates of abundance

While abundance estimates of individual colonies are variable using Landsat retrievals, our model for estimating abundance from the number of pixels identified as guano appears unbiased and thus neither consistently overpredicts or underpredicts abundance. Consequently, abundance estimates using Landsat retrievals improve at larger scales of aggregation. We find that abundance estimates using Landsat retrievals are statistically indistinguishable from those obtained using very-high resolution methods for aggregations containing >c. 40 colonies.

## Discussion

As described below, the retrievals of Adélie colony extent from medium-resolution imagery correlate well with comparable datasets, and exhibit relatively low errors of omission and commission. As has been demonstrated also for high-resolution imagery [4], the retrievals of colony extent (i.e. guano area) as captured by automated classification of Landsat imagery correlate well with abundance. As a result, this area-abundance relationship can be used as the basis for estimating abundance in areas where the population is otherwise unknown. As illustrated in Figure 1, the performance of the Landsat retrieval for colony detection improved with increasing colony population size. While nearly 100% of the smallest Adélie penguin colonies were undetected by the Landsat method, these contribute very little to regional and continental aggregated abundance and the Landsat retrievals are thus able to capture the vast majority of the Adélie penguin population. The Antarctic Peninsula has a significantly larger proportion of small colonies when compared to the rest of the continent and a higher threshold for detection, and consequently the Landsat method missed a larger number of these colonies in both absolute numbers and in proportion to the total.

### The Antarctic Peninsula remains a challenge

The higher threshold for colony detection on the Antarctic Peninsula likely stems from several factors. These include a larger ratio of exposed surface material compared to snow cover, a greater variability of rock and soil types compared to the rest of the continent, and increased surface weathering. The AP also experiences significantly more rainfall than the continental portions of Antarctica. Weathering may wash away guano making it more difficult to detect smaller colonies, and it may contribute to estimation errors as the guano stain grows or shrinks within a given breeding season. At the same time, the topography of the Antarctic Peninsula is highly complex, with steep terrain that may obscure smaller colonies. Finally, higher cloud cover results in fewer usable scenes each year.

One of the major challenges of using satellite imagery to map Adélie penguin colonies on the Antarctic Peninsula is the existence of two other, closely related, species of penguins (gentoo and chinstrap) whose guano displays similar spectral signatures to that of the Adélie. Moreover, these three species frequently nest in mixed-species colonies, and while differentiation of species has been shown possible in high-resolution imagery [9], the spatial resolution of Landsat is larger than the scale at which Adélie penguins may cluster within a mixed-species colony. In other words, a single Landsat pixel of a mixed-species colony likely contains multiple species that cannot be distinguished. Other *Pygoscelis* spp. penguins are not the only source of errors of commission. Flying bird species, such as the blue-eyed shag (*Phalacrocorax atriceps*), also produce guano that can be incorrectly identified as penguin guano and can cause errors of commission in both Landsat and VHR imagery.

### Errors of commission vary across regions and sensors

Our experience with both VHR and Landsat imagery interpretation has shown that the causes of false positives (errors of commission) are both region and sensor specific. In the Antarctic Peninsula region, errors of commission can stem from breeding blue-eyed shags, as well as from large areas of snow algae, which is often red or pink in color, and iron-tinted scree on steep sloping ridges (Lynch Unpublished data). As well, areas used by penguins for molting can contain significant accumulations of guano and confuse mapping of breeding territories. Landsat imagery retrievals may contain areas used by flying birds either alone or with breeding Adélie penguins. We have also found abiotically-driven errors of commission in Landsat retrievals due to large wet alluvial fans, and others apparently due to sediment runoff or chlorophyll in the water just offshore.

### Future research

Increased weathering, complex terrain, and the existence of congeneric species make it difficult to estimate omission and commission error rates for the Antarctic Peninsula. While a Landsat retrieval of a gentoo colony is an error of commission in the context of an Adélie survey, it still has biological significance, particularly if it identifies a colony that was previously undiscovered. The use of prior information regarding the distribution of penguins and the Antarctic's flying bird species may allow us to use adaptive classification methods that minimize both errors of commission and omission by essentially ‘raising the bar’ for the detection of previously unknown Adélie penguin colonies and simultaneously ‘lowering the bar’ for the re-detection of known Adélie penguin colonies. In the same vein, prior estimates (e.g., from direct field surveys) of the fraction of penguins that are of each species (Adélie, gentoo, and chinstrap) at mixed colonies may allow us to estimate Adélie abundance to produce less biased estimates at the regional of continental scale.

While Landsat imagery lacks the sensitivity to detect small colonies, and is therefore inappropriate for studying processes such as colonization and range expansion, we have demonstrated that it can be used to obtain estimates of Adélie abundance over regional scales, and that bias in regional estimates can be corrected for non-detection. Corrections for non-detection will be made more accurate by better statistical models for colony size, but the existing precision of regional and global Adélie abundance is minimally affected by non-detection of the smallest colonies.

## Supporting Information

Figure S1 **Penguin Point satellite imagery and photograph.**
(DOC)

Table S1 **Raw data used for the detection and abundance estimation modeling.**
(XLSX)

Table S2 **Details of the Landsat-7 imagery classification.**
(DOCX)

Table S3 **Locations and estimated abundance at previously unreported colonies.**
(DOC)
