# Supplementary material for: Mapping the Abundance and Distribution of Adélie Penguins Using Landsat-7: First Steps towards an Integrated Multi-Sensor Pipeline for Tracking Populations at the Continental Scale
Source: PLoS One. 2014 Nov 20;9(11):e113301. doi: 10.1371/journal.pone.0113301 (PMC4239023; doi:10.1371/journal.pone.0113301)

**Figure S1: Penguin Point satellite imagery and photograph.** (A) High-resolution satellite image (Worldview-2) of Penguin Point (5 February 2011). Copyright (2014) by DigitalGlobe, Inc. (B) Landsat-7 image of Penguin Point (24 November 2001). (C) Photograph of part of the penguin colony at Penguin Point (2 December 2006; Credit: Louise Blight).


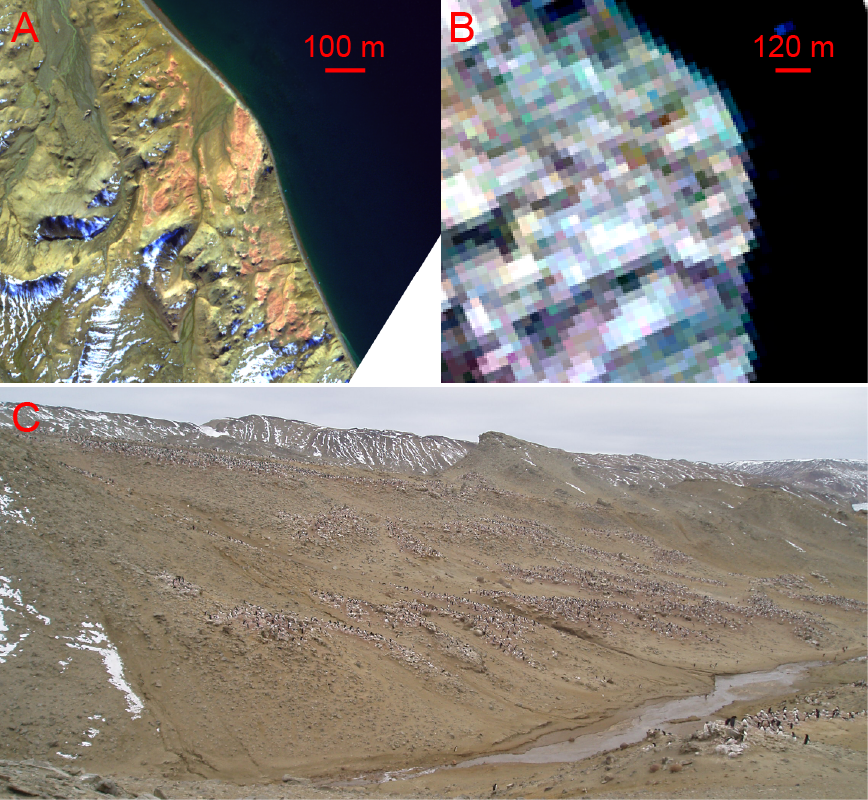

Supplement: Figure S1 — Penguin Point satellite imagery and photograph. (DOC) [file pone.0113301.s001.doc]
