# Supplementary material for: Mapping the Abundance and Distribution of Adélie Penguins Using Landsat-7: First Steps towards an Integrated Multi-Sensor Pipeline for Tracking Populations at the Continental Scale
Source: PLoS One. 2014 Nov 20;9(11):e113301. doi: 10.1371/journal.pone.0113301 (PMC4239023; doi:10.1371/journal.pone.0113301)
Supplement: Table S2 — Details of the Landsat-7 imagery classification. (DOCX) [file pone.0113301.s003.docx]

Supporting Information S1

Landsat Retrieval Methods

Following the methods described in [2], Landsat-7 ETM+ top of the atmosphere (TOA) reflectance data were processed to determine if any given pixel fell into the “Adélie penguin colony” class. Pixels were assigned to this class if their Euclidian distance, *d*, in transformed spectral space fell between 0 and 1. Here *d* was calculated as the square root of the sum of squares of the first 5 elements of the matrix **A**. The matrix, **A**, is calculated as follows.

$$\boldsymbol{A}= \boldsymbol{V}^{\boldsymbol{T}} \boldsymbol{・}\boldsymbol{T}^{\boldsymbol{-1}}$$

The transition matrix, **T**, was generated for the 6 ETM+ reflective bands from a training dataset. **T** =

$$\left[ \begin{matrix} 0.030515508 & 0.0055868545 & 0.10589161 & -0.017384585 & -0.0019109183 & 0.81610609 \\ 0.072376601 & 0.037022008 & 0.044094722 & 0.024166647 & 0.011383459 & 0.90759515 \\ 0.11551865 & 0.061577496 & -0.042806491 & -0.0088966133 & -0.011873039 & 0.90552486 \\ 0.10955417 & -0.076527096 & -0.019619026 & -0.0055267772 & 0.012877894 & 1.0822658 \\ 0.031201115 & -0.050623167 & 0.021522689 & 0.013288178 & -0.025795647 & 1.3335928 \\ 0 & 0 & 0 & 0 & 0 & 1 \end{matrix} \right]$$

The vector **V** = $\left[ \begin{matrix} \phi_{1} & \phi_{2} & \phi_{3} & \phi_{4} & \phi_{5} & 1 \end{matrix} \right]$ where each $\phi_{i}$ is constructed as follows.

$$\phi_{1}=arctan\left( \frac{\rho_{1}}{\rho_{2}} \right)$$

$$\phi_{2}=arctan\left( \frac{\rho_{1}+ \rho_{2}}{\rho_{3}} \right)$$

$$\phi_{3}=arctan\left( \frac{\rho_{1}+ \rho_{2}+\rho_{3}}{\rho_{4}} \right)$$

And so on, and where each $\rho_{i}$ = TOA reflectance for the 6 ETM+ reflective bands 1-5 and band 7. The retrieval results for the Antarctic Peninsula were aggregated with previous results for the southern continent and saved as a gzip-compressed Keyhole Markup Language (kmz) formatted file, which can be obtained at this url:

<ftp://trmm-fc.gsfc.nasa.gov/wolff/MattS/landsat_adelie_colonies_full_continent.kmz>.

Note that the retrieved Adélie penguin colony location and spatial extent stored in these files does not include colonies on the South Shetland or South Orkney Islands as these areas were cloud covered in the imagery available for the era studied. In addition, no imagery was available for Balleny Islands or Peter I Island.

Table S2. List of Adélie penguin colonies not included in the modeling of abundance. Latitude and Longitude reported in degrees. L7 = Landsat-7

| Location | Latitude | Longitude | Reason for exclusion from the abundance model |
| --- | --- | --- | --- |
| Kirby Head | -67.2726 | 46.5372 | L7 and VHR locations separated by several kilometers |
| Franklin Island West | -76.1618 | 168.3383 | Covered by shadow in L7 imagery |
| Hokuro Cove | -69.1973 | 39.6144 | L7 and VHR locations separated by several kilometers |
| South Svenner | -69.1341 | 76.7442 | Southwell (*Pers. Comm*.) suggests a larger abundance than found by [5]; true abundance of this population uncertain |
| Lovill Bluff | -73.5380 | -127.4361 | L7 retrieval likely includes a large colony of flying seabirds |
| Mount Biscoe | -66.2209 | 51.3275 | L7 retrieval likely includes a large colony of flying seabirds |
| Scullin/ Murray Monoliths | -67.7833 | 66.6968 | L7 retrieval likely includes a large colony of flying seabirds |
