# Supplementary material for: Mapping the Abundance and Distribution of Adélie Penguins Using Landsat-7: First Steps towards an Integrated Multi-Sensor Pipeline for Tracking Populations at the Continental Scale
Source: PLoS One. 2014 Nov 20;9(11):e113301. doi: 10.1371/journal.pone.0113301 (PMC4239023; doi:10.1371/journal.pone.0113301)
Supplement: Table S3 — Locations and estimated abundance at previously unreported colonies. (DOC) [file pone.0113301.s004.doc]

**Table S3: Locations and estimated abundance at previously unreported colonies.** Note that locations on the Antarctic Peninsula could contain gentoo, chinstrap, or Adélie penguins, and the abundance represents the total number of penguins of any species breeding at the site.

| Name | Region | Latitude | Longitude | # pixels | Estimated abundance  (95^th^ percentile CI) |
| --- | --- | --- | --- | --- | --- |
| − | Continent | -73.969 | -104.137 | 1 | 301 (255, 356) |
| − | Continent | -73.822 | -102.940 | 28 | 8508 (7427, 9652) |
| − | Continent | -73.663 | -101.522 | 14 | 4260 (3784, 4823) |
| − | Continent | -69.187 | 39.714 | 21 | 6388 (5660, 7281) |
| − | Continent | -67.455 | 60.882 | 13 | 3971 (3519, 4499) |
| − | Continent | -69.273 | 76.833 | 1 | 305 (257, 359) |
| − | Continent | -69.148 | 77.269 | 47 | 14325 (12578, 16127) |
| − | Continent | -66.368 | 110.453 | 1 | 305 (256, 359) |
| Bruce Island | Peninsula | -64.895 | -63.116 | 2 | 931 (681, 1238) |
| − | Peninsula | -64.891 | -63.943 | 1 | 465 (340, 643) |
| − | Peninsula | -64.201 | -60.982 | 5 | 2348 (1741, 3127) |
| Earle Island | Peninsula | -63.487 | -54.787 | 51 | 23649 (17361, 32163) |
| Darwin Island | Peninsula | -63.432 | -54.736 | 16 | 7419 (5384, 9931) |
| Brash Island | Peninsula | -63.385 | -54.915 | 359 | 166078 (123666, 228268) |
| Demas Rocks-2 | Peninsula | -63.341 | -58.046 | 6 | 2778 (2086, 3798) |
| Pelusa Point | Peninsula | -63.313 | -57.909 | 17 | 7898 (5799, 10626) |
| − | Peninsula | -63.027 | -56.008 | 3 | 1400 (1000, 1918) |
| − | Peninsula | -62.997 | -56.022 | 1 | 463 (339, 638) |
| near d'Urville Island | Peninsula | -62.995 | -56.273 | 6 | 2774 (2039, 3719) |
| − | Peninsula | -62.994 | -55.978 | 2 | 914 (664, 1261) |
| Stoker Island | Peninsula | -62.397 | -59.849 | 16 | 7360 (5552, 10169) |
| − | Peninsula | -62.372 | -59.781 | 1 | 467 (337, 644) |
| − | Peninsula | -62.370 | -59.761 | 3 | 1405 (1033, 1908) |
| Catharina Point | Peninsula | -62.329 | -59.620 | 5 | 2316 (1686, 3166) |
| − | Peninsula | -61.984 | -58.541 | 1 | 464 (332, 636) |
